# Prenatal exposure to fine particulate matter and newborn anogenital distance: a prospective cohort study

**DOI:** 10.1186/s12940-023-00969-w

**Published:** 2023-02-09

**Authors:** Xiaoli Shen, Xia Meng, Cuiping Wang, Xiangfeng Chen, Qian Chen, Jing Cai, Jun Zhang, Qianlong Zhang, Lichun Fan

**Affiliations:** 1 Ministry of Education-Shanghai Key Laboratory of Children’s Environmental Health, Xinhua Hospital, Shanghai Jiao Tong University School of Medicine, Shanghai, China; 2 School of Public Health, Shanghai Jiao Tong University School of Medicine, Shanghai, China; 3 School of Public Health, Key Lab of Public Health Safety of the Ministry of Education and NHC Key Lab of Health Technology Assessment, Fudan University, Shanghai, 200032 China; 4 Center for Reproductive Medicine, Ren Ji Hospital, School of Medicine, Shanghai Jiao Tong University, Shanghai, 200135 China; 5 Shanghai Key Laboratory for Assisted Reproduction and Reproductive Genetics, Shanghai, 200135 China; 6 Shanghai Human Sperm Bank, Shanghai, 200135 China; 7 Women and Children’s Medical Center of Hainan Province, No.75, Longkunnan Road, Haikou, 570100 Hainan China

**Keywords:** Fine particulate matter, Anogenital distance, Reproductive development, Prenatal exposure

## Abstract

**Background:**

Considerable attention has been paid to reproductive toxicity of fine particulate matter (PM_2.5_). However, the relationship between prenatal PM_2.5_ exposure and anogenital distance (AGD) has not been well studied. We aim to investigate the potential effects of prenatal exposure to PM_2.5_ on newborn AGD.

**Methods:**

Prenatal PM_2.5_ exposure of 2332 participates in Shanghai (2013–2016) was estimated using high-performance machine learning models. Anoscrotal distance (AGDas) in male infants and anofourchette distance (AGDaf) in female infants were measured by well-trained examiners within 3 days after birth. We applied multiple linear regression models and multiple informant models to estimate the association between prenatal PM_2.5_ exposure and AGD.

**Results:**

Multiple linear regression models showed that a 10 μg/m^3^ increase in PM_2.5_ exposure during full pregnancy, the second and third trimesters was inversely associated with AGDas (adjusted beta = − 1.76, 95% CI: − 2.21, − 1.31; − 0.73, 95% CI: − 1.06, − 0.40; and − 0.52; 95% CI: − 0.87, − 0.18, respectively) in males. A 10 μg/m^3^ increase in PM_2.5_ exposure during the full pregnancy, the first, second, and third trimesters was inversely associated with AGDaf (adjusted beta = − 4.55; 95% CI: − 5.18, − 3.92; − 0.78; 95% CI: − 1.10, − 0.46; − 1.11; 95% CI: − 1.46, − 0.77; − 1.45; 95% CI: − 1.78, − 1.12, respectively) in females after adjusting for potential confounders. Multiple informant models showed consistent but slightly attenuated associations.

**Conclusion:**

Our study observed a significant association between gestational PM_2.5_ exposure during pregnancy and shortened AGD in newborns, and provided new evidence on potential reproductive toxicity of prenatal PM_2.5_ exposure.

**Supplementary Information:**

The online version contains supplementary material available at 10.1186/s12940-023-00969-w.

## Background

Fine particulate matter (particles with aerodynamic diameters of 2.5 μm or less, PM_2.5_) is often considered to be the major contributor to air pollution [1]. PM_2.5_ has small particles and large surface area, which makes it easy to penetrate into deeper respiratory tracts and even enter blood circulation [2, 3], resulting in severe threats to multiple body systems including respiratory [4], circulatory [5], central nervous [6] and reproductive systems [7]. According to the Global Burden of Disease Study 2019, PM_2.5_ ranks the seventh in all health risks and has caused 4.14 million deaths globally per year (1.42 million in China) [4].

Numerous studies suggested that PM_2.5_ may cause potential risks to the human reproductive system [7–10]. One recent review concluded that PM_2.5_ exposure may cause abnormal spermatogenesis, sperm malformation and disrupted hormone levels and, ultimately, infertility [7]. Emerging evidence suggests that the effects of PM_2.5_ can be traced back to the gestational period and the presence of fine particles in human placental tissue cells has been detected [11–13]. Moreover, epidemiological studies have demonstrated that maternal exposure of PM_2.5_ is associated with adverse birth outcomes, such as premature birth, stillbirth, and low birth weight [14–17]. However, studies directly linked the associations of early-life PM_2.5_ exposure with fetal development of reproductive system are still limited. Ren et al. reported that maternal PM_2.5_ exposure caused structural testicular lesions, decreased sperm quality and disrupted testosterone levels in offspring. Another study examined the reproductive toxicity of gestational exposure to traffic pollutants and found that such exposure induced abnormal spermatogenesis and altered genome-wide mRNA and microRNA expression in F2 male mice [18].

Anogenital distance (AGD), defined as the distance from the anus to the genitals, is thought to be a sensitive biomarker reflecting reproductive toxicity [19]. AGD is a sexually dimorphic trait and males have longer AGD than females [20]. Evidence from animal studies confirmed that reduced AGD in newborn may result from higher in utero anti-androgenic exposure [21, 22]. In males, it was suggested that shorter AGD is related to negative reproductive outcomes like cryptorchidism [23], hypospadias [24] and long-life disorders like impaired sperm quality [25, 26]. In females, AGD was suggested to be associated with endometriosis [27] and ovarian function [28] Thus, AGD can be used as another indicator for offspring’s reproductive system development and to predict late-life reproductive disorders for both genders. Moreover, several epidemiologic studies have revealed that prenatal exposure to endocrine disrupting chemicals was linked to altered AGD [19, 29–31]. To date, only one epidemiological study measured AGD in 876 mother-infant pairs in Shanghai, China, and explored the relationship between gestational PM_2.5_ exposure and newborn AGD. They revealed a negative association of prenatal PM_2.5_ exposure during first and third trimesters with AGD in both genders [32]. To obtain a further understanding of the potential early-life reproductive toxicity of PM_2.5_, we investigated the association of prenatal exposure to PM_2.5_ in different trimesters and newborn AGD in a multicenter prospective birth cohort study in Shanghai, China.

## Material and methods

### Study population

The Shanghai Birth Cohort (SBC) is a prospective study aiming to assess the potential health impacts of genetic, environmental, and behavioral factors on fertility, pregnancy outcomes and child growth. A detailed description of SBC can be found elsewhere [33]. To be eligible, women had to meet the following criteria: they were 20 years old and above, registered residents in Shanghai and had no plans to leave Shanghai within 2 years, planned to go to SBC collaborating hospitals for antenatal examination and delivery, and were willing to come for multiple follow-up visits lasting for at least 2 years. At the enrollment, the participants were asked to provide detailed information on sociodemographic characteristics, behaviors and lifestyles, environmental factors, and reproductive and medical history through questionnaire survey. An informed consent was signed by each participant and the study protocol was approved by the Ethics Committee of Xinhua hospital, Shanghai Jiao Tong University School of Medicine.

**Fig. 1:**
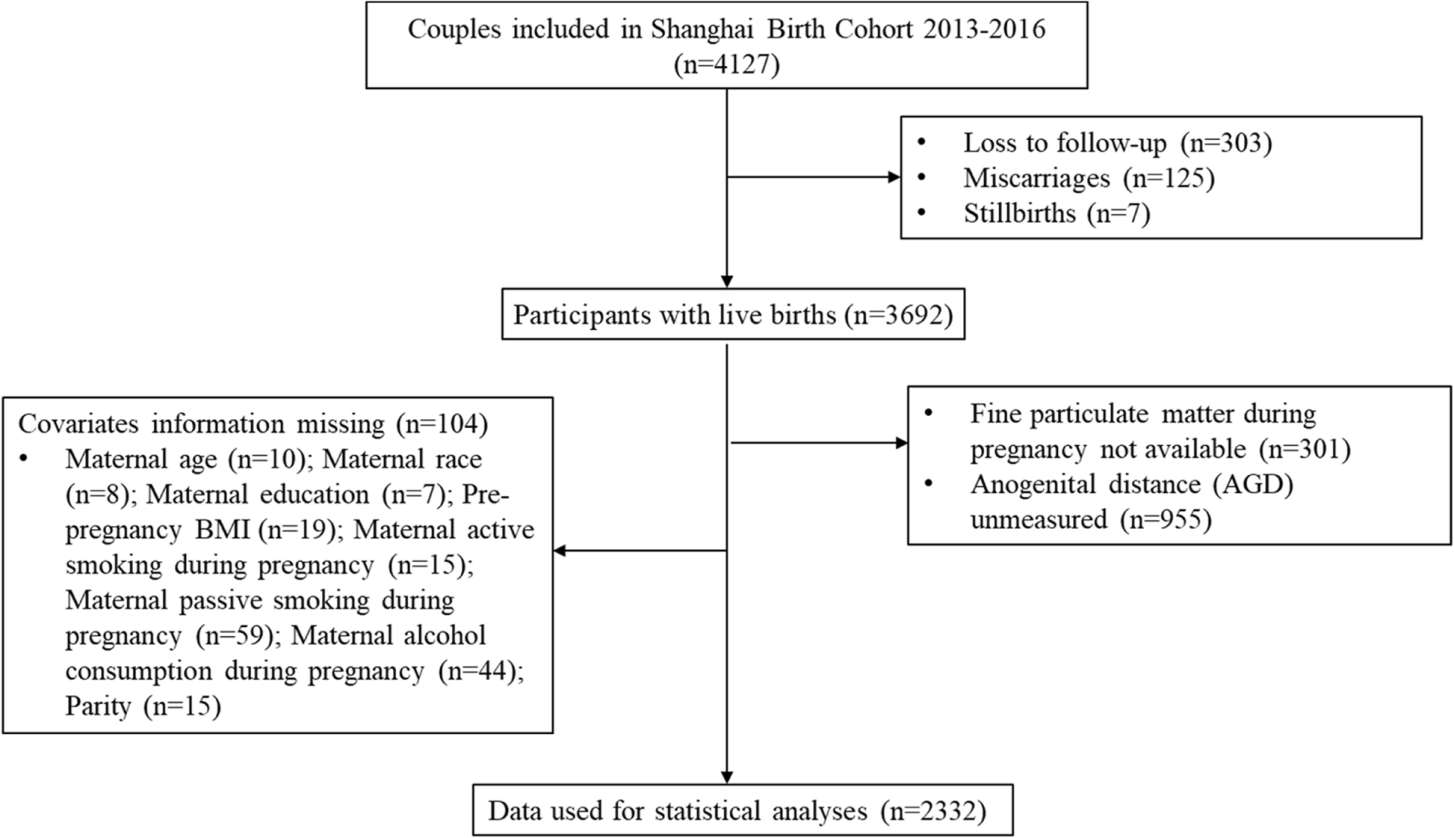
Flow chart of the selection procedure of study participants

From 2013 to 2016, a total of 4127 women were enrolled in the SBC. For the current analysis, we restricted to women who had PM_2.5_ exposure data available, whose infants’ AGD measured, and who had no missing data on major covariates. As a result, 2332 subjects were included (Fig. 1).

### PM_2.5_ exposure assessment

Each participant’s residential address was collected through self-reported questionnaire and then geocoded. Daily ambient PM_2.5_ levels of each participant were predicted using machine learning algorithms with high spatial resolutions (1 km × 1 km) developed previously [34]. Briefly, random forest algorithm was employed to develop models to predict PM_2.5_ concentrations with available aerosol optical depth (AOD) data on days and at grid cells or without AOD data separately. Other factors including meteorological data, land use information and population variables were also incorporated in the model to improve the accuracy. Cross-validation R^2^ between daily predictions and ground measurements was 0.81. Then, daily prediction of PM_2.5_ concentrations were assigned to participants according to their residential address. Then, the mean value was calculated to represent the PM_2.5_ level throughout the full pregnancy and specific trimesters. In our study, we defined first trimester as 1 to 13 gestational weeks, second trimester as 14 to 27 gestational weeks and third trimester as 28 gestational weeks to the birth.

### Anogenital distance measurement

Infants’ AGDs were measured by trained examiners within 3 days after birth. AGDas (from the center of the anus to the posterior base of the scrotum) were measured in male infants and AGDaf (from the center of the anus to the posterior fourchette) were measured in female infants following a standardized method described by Salazar-Martinez [20]. In brief, an infant was placed on a flat table on his back and his thigh was tightly flexed in order to completely expose the genitals and anus. Then, the examiner used a Vernier caliper that is precise to 0.1 mm to conduct the measurements. Two measurements were taken and if the differences between the two measurements were more than 2 mm, a third measurement was taken. For further analysis, we calculated the mean value of the two measurements; if a third measurement was taken, the mean of the two closest measurements was calculated. Moreover, examiners were not aware of the prenatal air pollution exposure levels of the infants.

### Covariates

Covariates were selected according to a directed acyclic graph (Fig. S1), previous literature and data accessibility. Since nearly all pregnant women were Han ethnicity (98.7%), never smoking (99.5%) or consuming alcohol (99.4%) during pregnancy, we did not consider these variables as potential confounders. Additionally, we excluded gestational age and birth weight in the final model as we assumed that they may be intermediators but not confounders between PM_2.5_ exposure and AGD according to previous literature [35, 36]. In the end, the covariates included in the analysis were maternal age (years), maternal education (below Bachelor, Bachelor, above Bachelor), pre-pregnancy body mass index (BMI, kg/m^2^), maternal passive smoking during pregnancy (Yes, No), parity (Nulliparous, Multiparous) and birth season (Spring, Summer, Autumn, Winter). We derived maternal covariates via questionnaires during pregnancy and extracted infant information from medical records.

### Statistical analysis

The distribution of the newborn AGD and sociodemographic characteristics using frequencies (proportions) for categorical variables and means (standard deviations, SD) for continuous variables were described among all subjects and different sex separately. The distribution of the PM_2.5_ exposure levels across the entire pregnancy period and specific trimester were also described.

Multiple linear regression models were applied to demonstrate the associations of PM_2.5_ exposure (continuous) with newborn AGD during specific trimester and entire pregnancy, and fitted unadjusted and adjusted models separately. We also analyzed the potential non-linear association by categorizing the PM_2.5_ exposure into quartiles and set the lowest quartile as the reference level adjusting for the same covariates. Then, a multiple informant model with generalized estimating equation was additionally applied [37]. This model took advantage of repeated PM_2.5_ measurements for three trimesters. We added the interaction terms between PM_2.5_ and trimesters to test whether the estimates between PM_2.5_ exposure and AGDs differed across trimesters to determine the vulnerable periods. Finally, we performed sensitivity analyses by: a) additionally adjusting for gestational age and birth weight; b) using the multiple imputation to generate five imputed datasets without missing covariates data and obtained the pooled estimates following the Rubin’s rules [38–40]; c) excluding infants who were low birth weight (< 2500 g) or macrosomia (> 4000 g). Variance Inflation Factor (VIF) was calculated to evaluate the collinearity among independent variables. All analyses were conducted using R version 3.5.1 and the multiple imputation was conducted by R package “mice”.

## Results

Table 1 shows the distribution of the sociodemographic characteristics and AGDs. Among all the 2332 subjects, the average maternal age was 28.5 years old and the majority of them were nulliparous (85.2%). Most of the pregnant women were well-educated and had a normal BMI before pregnancy (pre-pregnancy BMI among 18.5–23.9). Almost all pregnant women never smoked (99.5%) or drank alcohol (99.4%) during pregnancy while 40.6% were exposed to passive smoking during pregnancy. The mean AGD was 18.7 mm in male and 11.1 mm in female infants. The socio-demographic characteristics between the included and excluded women differed in maternal education, maternal pre-pregnancy BMI, maternal age, birth season, gestational age and infant length (*P* < 0.05). There were no statistically significant differences in other characteristics (e.g., anogenital distance and birthweight) (Table S1).

**Table 1:** Characteristics of mother-infant pairs in the analysis in the Shanghai Birth Cohort

Variable	Male	Female
	N = 1186	N = 1146
Maternal race [N (%)]		
Han ethnicity	1174 (99.0)	1128 (98.4)
Other	12 (1.0)	18 (1.6)
Maternal Education [N (%)]		
<Bachelor	439 (37.0)	390 (34.0)
Bachelor	612 (51.6)	597 (52.1)
Graduate and above	135 (11.4)	159 (13.9)
Pre-pregnancy BMI (kg/m^2^) [N (%)]		
< 18.5	178 (15.0)	168 (14.7)
18.5–23.9	827 (69.7)	788 (68.8)
≥24	181 (15.3)	190 (16.6)
Maternal active smoking during pregnancy [N (%)]		
No	1181 (99.6)	1139 (99.4)
Yes	5 (0.4)	7 (0.6)
Maternal passive smoking during pregnancy [N (%)]		
No	699 (58.9)	686 (59.9)
Yes	487 (41.1)	460 (40.1)
Maternal alcohol consumption during pregnancy [N (%)]		
No	1177 (99.2)	1142 (99.7)
Yes	9 (0.8)	4 (0.3)
Parity [N (%)]		
Nulliparous	1020 (86.0)	966 (84.3)
Multiparous	166 (14.0)	180 (15.7)
Birth season [N (%)]		
Spring	248 (20.9)	197 (17.2)
Summer	309 (26.1)	352 (30.7)
Autumn	403 (34.0)	367 (32.0)
Winter	226 (19.1)	230 (20.1)
Maternal Age (years) [Mean (SD)]		
	28.6 (3.7)	28.5 (3.6)
Gestational age (weeks) [Mean (SD)]		
	38.9 (1.4)	39.1 (1.3)
Birth weight (g) [Mean (SD)]		
	3411 (432)	3325 (408)
Length (cm) [Mean (SD)]		
	50.0 (1.2)	49.8 (1.1)
Anogenital distance (mm) [Mean (SD)]		
	18.7 (4.0)	11.1 (3.8)

*Abbreviations*: *SD* standard deviation

The distribution of the average level of PM_2.5_ exposure is displayed in Table 2. The average level of PM_2.5_ exposure in all participants was 50 μg/m^3^ during the full pregnancy and 53 μg/m^3^, 49 μg/m^3^, 48 μg/m^3^ in the first, second and third trimesters, respectively.

**Table 2:** Average PM_2.5_ levels (μg/m^3^) during pregnancy in Shanghai Birth Cohort (*N* = 2332)

	All subjects N = 2332		Male N = 1186		Female *N* = 1146	
Period	Mean (SD)	Median (IQR)	Mean (SD)	Median (IQR)	Mean (SD)	Median (IQR)
Full pregnancy	50 (5)	50 (7)	50 (5)	50 (7)	50 (5)	50 (7)
First trimester	53 (13)	51 (23)	52 (13)	51 (22)	53 (13)	52 (23)
Second trimester	49 (13)	45 (17)	49 (13)	45 (18)	49 (12)	45 (17)
Third trimester	48 (12)	45 (18)	48 (12)	45 (18)	48 (13)	43 (18)

*Abbreviations*: *PM*_*2.5*_ particles with aerodynamic diameters of 2.5 μm or less, *SD* standard deviation, *IQR* interquartile range

Table 3 shows the unadjusted and adjusted associations between maternal PM_2.5_ exposure and AGDs from multiple linear regression model. In the unadjusted models, PM_2.5_ showed generally inverse associations with AGDs. After adjusting for maternal age, maternal education, pre-pregnancy BMI, maternal passive smoking, parity and birth season, a 10 μg/m^3^ increase in PM_2.5_ exposure across the full pregnancy was negatively associated with AGDas in male infants (adjusted beta = − 1.91; 95% CI: − 2.57, − 1.24) and more strongly and negatively associated with AGDaf in female infants (adjusted beta = − 4.55; 95% CI: − 5.18, − 3.92).

**Table 3:** Relationship between maternal exposure to PM_2.5_ per 10 μg/m^3^ and offspring’s anogenital distance in multiple linear regression models

	Model 1^a^		Model 2^a^	
	β (95%CI)	*P* value	β (95%CI)	*P* value
Male (*N* = 1186)				
Full pregnancy	**−1.76 (−2.21, − 1.31)**	**< 0.001**	**− 1.91 (− 2.57, − 1.24)**	**< 0.001**
First trimester	**0.28 (0.10, 0.45)**	**0.002**	−0.15 (− 0.46, 0.17)	0.353
Second trimester	**− 0.54 (− 0.72, − 0.36)**	**< 0.001**	**−0.73 (− 1.06, − 0.40)**	**< 0.001**
Third trimester	**− 0.59 (− 0.77, − 0.40)**	**< 0.001**	**− 0.52 (− 0.87, − 0.18)**	**< 0.001**
Female (*N* = 1146)				
Full pregnancy	**−2.04 (− 2.50, − 1.59)**	**< 0.001**	**− 4.55 (− 5.18, − 3.92)**	**< 0.001**
First trimester	−0.14 (− 0.31, 0.03)	0.106	**− 0.78 (− 1.10, − 0.46)**	**< 0.001**
Second trimester	**−0.29 (− 0.47, − 0.11)**	**0.002**	**− 1.11 (− 1.46, − 0.77)**	**< 0.001**
Third trimester	**−0.44 (− 0.62, − 0.27)**	**< 0.001**	**− 1.45 (− 1.78, − 1.12)**	**< 0.001**

*Abbreviations*: *PM*_*2.5*_ particles with aerodynamic diameters of 2.5 μm or less, *95% CI* 95% confidence interval

^a^Model 1 was unadjusted; Model 2 was adjusted for maternal age, maternal education, pre-pregnancy BMI, maternal passive smoking during pregnancy, parity and birth season

With regard to the trimester specific exposure, in the first trimester, the inverse relationship with AGDas in male infants was nonsignificant (adjusted beta = − 0.15; 95% CI: − 0.46, 0.17) but statistically significant with AGDaf in female infants (adjusted beta = − 0.78; 95% CI: − 1.10, − 0.46). In the second and third trimesters, we observed consistent and stronger negative associations in both genders. For males, each 10 μg/m^3^ increase in PM_2.5_ was associated with 0.73 mm decrease in AGDas in the second trimester and 0.52 mm decrease in the third trimester. For females, each 10 μg/m^3^ increase in PM_2.5_ was associated with 1.11 mm decrease in AGDaf in the second trimester and 1.45 mm decrease in the third trimester.

**Fig. 2:**
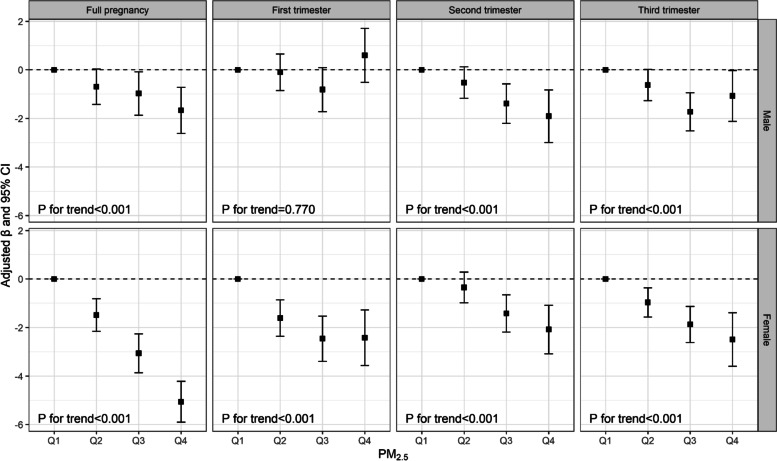
Relationship between maternal exposure to PM_2.5_ (μg/m^3^) (in quartiles) and offspring’s anogenital distances (mm). Multiple linear regression models were adjusted for maternal age, maternal education, pre-pregnancy BMI, maternal passive smoking during pregnancy, parity and birth season

The relationship between maternal PM_2.5_ exposure (in quartiles) and offspring’s AGDs was illustrated in Fig. 2 and the detailed estimates were listed in Table S2. Similarly, gestational exposure to PM_2.5_ throughout pregnancy showed decreasing linear associations with AGD. PM_2.5_ exposure in the highest quartile was linked to 1.67 (95% CI: − 2.62, − 0.72) mm decrease in AGDas in male infants and 5.06 (− 5.90, − 4.21) mm decrease in AGDaf in female infants across the full pregnancy and the test for trend was significant. The significant inverse relationship was also observed in the first trimester in female infants and in the second and third trimesters in both genders.

The results from the multiple informant models are shown in Table 4. We observed consistent but weaker associations compared to those shown in Table 3. In male infants, each 10 μg/m^3^ increase in PM_2.5_ was correlated with a significant decrease in AGDas during second trimester (adjusted beta = − 0.27; 95% CI: − 0.40, − 0.14) and third trimester (adjusted beta = − 0.20; 95% CI: − 0.34, − 0.05) and for female infants increasing levels of PM_2.5_ was statistically significantly correlated with decreasing AGDaf during all trimesters. We assessed the VIF for all the regression models and the values were all less than 5, indicating that there was no collinearity among independent variables.

**Table 4:** Relationship between maternal exposure to PM_2.5_ per 10 μg/m^3^ and offspring’s anogenital distance in multiple informant models

Male (*N* = 1186)	β (95%CI) ^a^	*P* value
First trimester	0.01 (− 0.13, 0.15)	0.899
Second trimester	**−0.27 (− 0.40, − 0.14)**	**< 0.001**
Third trimester	**− 0.20 (− 0.34, − 0.05)**	**0.008**
Female (*N* = 1146)		
First trimester	**−0.15 (− 0.28, − 0.02)**	**0.028**
Second trimester	**− 0.37 (− 0.50, − 0.25)**	**< 0.001**
Third trimester	**−0.41 (− 0.54, − 0.29)**	**< 0.001**

*Abbreviations*: *PM*_*2.5*_ particles with aerodynamic diameters of 2.5 μm or less, *95% CI* 95% confidence interval

^a^Multiple informant model was adjusted for maternal age, maternal education, pre-pregnancy BMI, maternal passive smoking during pregnancy, parity and birth season

The results from our sensitivity analysis were generally similar to our main findings (Tables S3-S5). When we additionally adjusted for gestational age and birthweight, using data with multiple imputation and excluding infants who were low birth weight or macrosomia, significantly inverse associations of maternal PM_2.5_ exposure with AGDs still existed in the second and third trimesters in male infants and in all trimesters in female infants.

## Discussion

Our large prospective study (*n* = 2332) showed that prenatal exposure to PM_2.5_, especially in the second and third trimesters, was negatively correlated with neonatal AGD after controlling for potential confounders. This study adds more epidemiological evidence regarding the potential productive toxicity of in utero PM_2.5_ exposure.

Numerous studies have been carried out to investigate the health impact of PM_2.5_ on the reproductive system [41–44]. In males, it has been found in rodent studies that high concentrations of PM_2.5_ could cause abnormal spermatogenesis and destroy the blood-testis barrier integrity, leading to decreased semen motility [41]. Another research in mice reported that PM_2.5_ could also induce semen DNA double strand breaks and influence the semen quality [8]. In females, studies have shown that PM_2.5_ exposure could cause decline in ovarian reserve and induce adverse perinatal outcomes [7]. Moreover, PM_2.5_ was shown to be related to decreased circulating concentrations of testosterone and follicle-stimulating hormone, indicating harmful effects of PM2.5 on hormone concentrations [37]. Since AGD is mainly determined by in utero androgen levels and can be utilized to predict late-life reproductive health, the studies mentioned above support our findings.

However, studies directly link gestational PM_2.5_ exposure to neonatal reproductive system are still limited. Several animal studies explored the hazards of early-life PM_2.5_ exposure on the reproductive function of the offspring. Ren et al.  reported that in utero PM_2.5_ may induce testicular cell apoptosis and declining testosterone secretion prompted by the UPR-mediated JNK pathway in mice. Another study looked at the consequences of maternal exposure of traffic pollutants (included PM_2.5_) and the findings indicated that gestational exposure to traffic pollutants may impair spermatogenic function through disrupting the testicular immune environment via the abnormal miRNA and mRNA expression in F2 male mice [18].

So far, only one epidemiologic study examined the association between prenatal PM_2.5_ exposure and AGD. Sun et al. (2020) measured AGD in 876 mother-infant pairs in Shanghai, China, and reported that the mean AGDas in males was 15.5 mm and mean AGDaf in females was 9.5 mm, which were close to ours (AGDas: 18.7 mm, AGDaf: 11.1 mm). The average PM_2.5_ levels throughout the full pregnancy in Shanghai-Minhang birth cohort was 62.8 μg/m^3^, somewhat higher than 49.84 μg/m^3^ in our study. They identified a statistically significant negative relationship for PM_2.5_ throughout pregnancy and neonatal AGDas in male infants (beta = − 0.439, 95%CI: − 0.678, − 0.200) and AGDaf in female infants (beta = − 0.306, 95%CI: − 0.588, − 0.023). Their findings were quite consistent with ours. Both studies were conducted in Shanghai, which lent the results more comparable. However, most of the participants received higher levels of education and had relatively high income, making the results not representative of less developed regions. Therefore, more epidemiological studies are required to further investigate the reproductive toxicity of early-life PM_2.5_ exposure.

The underlying biological mechanisms of the relationship between prenatal PM_2.5_ exposure and AGD remain unclear. It is plausible that PM_2.5_ may affect AGD through disrupting thyroid hormone status. A Belgium birth cohort study indicated that exposure to PM_2.5_ during late pregnancy was significantly and inversely correlated with TSH levels in the cord blood [45]. Another study in Shanghai also observed a negative relationship between PM_2.5_ and FT4 in maternal serum ([45, 46]. Lower cord serum FT4 and TSH levels were negatively correlated with shorter AGD in male newborns [47], suggesting that the disruption of thyroid function may be a potential cause for shortened AGD.

Additionally, PM_2.5_ may absorb endocrine-disrupting chemicals (EDCs) such as phthalate esters, bisphenol A, alkylphenols and natural and synthetic sex hormones that may exert estrogenic and anti-androgenic activities [48, 49]. As AGD is considered as an androgen-sensitive biomarker, we hypothesize that these EDCs in PM_2.5_ may act as androgen receptor antagonists and influence the AGD length in offspring. Numerous studies have demonstrated that early-life EDC exposure has a negative impact on AGD. For instance, a Swedish study found that higher prenatal phthalates exposure in first trimester was related to shorter AGDas in males [29]. Another study from Mexico also observed the reduction of AGD with a higher index of maternal phthalates exposure in male offspring [30]. As for females, Mammadov et al. found that prenatal higher bisphenol A exposure was linked to shorter AGDas in females [50]. Moreover, a couple of research investigated maternal EDC mixture exposure effects on AGD. One recent Spanish study assessed the joint effects of 18 persistent organic pollutants on AGD in 129 children and found that co-exposure to persistent organic pollutants during pregnancy was associated with a reduced AGD in males but not in females [51]. Interestingly, another study from Shanghai, China, demonstrated that exposure to perfluoroalkyl substances mixture during pregnancy was associated with increased AGD in female neonates [52].

There has been a debate on which critical exposure window PM_2.5_ affects AGD in offspring. Our study did not observe specific vulnerable periods in females. We speculated that middle to late pregnancy might be more sensitive for AGD because testosterone production occurs between 8 to 37 weeks of gestation in fetal and, thus, impact the reproductive tract development [53]. Further, the length of AGD may be affected by fetal growth [54]. PM_2.5_ was found to have an adverse impact on birthweight during the third trimester [55–57]. However, an animal study suggested that the programming window for all male reproductive tract development in humans might be 8 to14 weeks of gestation, during which period AGD was more responsive [58]. Besides, Sun et al. [32] reported that both the first and third trimesters were sensitive periods for the effect of PM_2.5_ on AGD in their cohort study. Further exploration of the sensitive exposure window of PM_2.5_ are warranted.

Our study has several strengths. It was a large prospective cohort study that could clearly demonstrate a temporal relationship between exposure and outcome, reduce selection bias and recall bias, and have adequate statistical power. In addition, we adopted the machine learning algorithms to predict PM_2.5_ levels for each participant at high resolutions (1 × 1 km). This method showed high predictive ability and made the PM_2.5_ exposure proxy more precise than the data acquired from nearest monitoring station.

However, our study also has some limitations. First, we predicted the PM_2.5_ value for each participant based on her residential address. This value may not be an accurate proxy. Since women in our cohort mostly had jobs and did not stay at home all the time during their pregnancy, misclassification of the exposure level was inevitable. More research is warranted to confirm or refute our findings. Moreover, PM_2.5_ is a complicated mixture. It contains various components, including heavy metals, elemental carbon, organic chemicals and acids [3, 59]. We only measured the total PM_2.5_ concentrations and did not further assess the hazards of its different components. Additionally, residual confounding by measured or unmeasured variables is still possible. There was possible inter-examiner variability in AGD measurements due to the involvement of multiple examiners in the study, despite that we had tried our best to standardize the measurement by rigorous training.

## Conclusion

Our prospective cohort study revealed that prenatal exposure to PM_2.5_, especially during the second and third trimesters, was negatively associated with AGD in infants. Our findings highlight the importance to protect pregnant women from effects of PM_2.5_ exposure, especially those in high polluted areas. Further studies are needed to identify the life-long reproductive hazards of PM_2.5_ and possible biological mechanisms.

## Supplementary Information

**Additional file 1: Table S1.** Characteristics of included and excluded mother-infant pairs in the Shanghai Birth Cohort. **Table S2.** Relationship between maternal exposure to PM_2.5_ (μg/m^3^) (in quartiles) and offspring’s anogenital distances (mm). **Table S3.** Sensitivity analysis for the relationship between maternal exposure to PM_2.5_ per 10 μg/m^3^ and offspring’s anogenital distances (mm) additionally adjusted for gestational age and birth weight. **Table S4.** Sensitivity analysis for the relationship between maternal exposure to PM_2.5_ per 10 μg/m^3^ and offspring’s anogenital distances (mm) based on the data with multiple imputation. **Table S5.** Sensitivity analysis for the relationship between maternal exposure to PM_2.5_ per 10 μg/m^3^ and offspring’s anogenital distances (mm) based on the data excluding low birth weight and high birth weight.

**Additional file 2: Figure S1.** Directed Acyclic Graph for covariates selection.

## Data Availability

The dataset generated and/or analyzed in the current study are not publicly available but can be obtained from the corresponding author on a reasonable request.

## Abbreviations

BMI: Body mass index
CI: Confidence interval
PM_2.5_: Particles with aerodynamic diameters of 2.5 μm or less
AGD: Anogenital distance
AGDas: Anoscrotal distance
AGDaf: Anofourchette distance
SBC: Shanghai Birth Cohort
