# Supplementary material for: Prenatal exposure to fine particulate matter and newborn anogenital distance: a prospective cohort study
Source: Environ Health. 2023 Feb 9;22:16. doi: 10.1186/s12940-023-00969-w (PMC9909868; doi:10.1186/s12940-023-00969-w)
Supplement: Supplementary file 1 — Additional file 1: Table S1. Characteristics of included and excluded mother-infant pairs in the Shanghai Birth Cohort. Table S2. Relationship between maternal exposure to PM2.5 (μg/m3) (in quartiles) and offspring’s anogenital distances (mm). Table S3. Sensitivity analysis for the relationship between maternal exposure to PM2.5 per 10 μg/m3 and offspring’s anogenital distances (mm) additionally adjusted for gestational age and birth weight. Table S4. Sensitivity analysis for the relationship between maternal exposure to PM2.5 per 10 μg/m3 and offspring’s anogenital distances (mm) based on the data with multiple imputation. Table S5. Sensitivity analysis for the relationship between maternal exposure to PM2.5 per 10 μg/m3 and offspring’s anogenital distances (mm) based on the data excluding low birth weight and high birth weight. [file 12940_2023_969_MOESM1_ESM.docx]

**Table S1. Characteristics of included and excluded mother-infant pairs in the Shanghai Birth Cohort.**

| Variable | Included  N=2332 |  | Excluded  N=1360 | *P* Value |
| --- | --- | --- | --- | --- |
| Maternal race [N (%)] | |  |  | 0.587 |
| Han ethnicity | 2302 (98.7) |  | 1328(98.4) |  |
| Other | 30 (1.3) |  | 21 (1.6) |  |
| Maternal Education [N (%)] | |  |  | **0.006** |
| <Bachelor | 829 (35.5) |  | 417 (31.1) |  |
| Bachelor | 1209 (51.8) |  | 718 (53.5) |  |
| Graduate and above | 294 (12.6) |  | 206 (15.4) |  |
| Pre-pregnancy BMI (kg/m^2^) [N (%)] | | | | **0.012** |
| <18.5 | 346 (14.8) |  | 241 (17.7) |  |
| 18.5-23.9 | 1615 (69.3) |  | 877 (64.6) |  |
| ≥24 | 371 (15.9) |  | 239 (17.6) |  |
| Maternal active smoking during pregnancy [N (%)] | | | | 0.501 |
| No | 2320 (99.5) |  | 1319 (99.7) |  |
| Yes | 12 (0.5) |  | 4 (0.3) |  |
| Maternal passive smoking during pregnancy [N (%)] | | | | 0.656 |
| No | 1385 (59.4) |  | 682 (58.5) |  |
| Yes | 947 (40.6) |  | 483 (41.5) |  |
| Maternal alcohol consumption during pregnancy [N (%)] | | | | 0.318 |
| No | 2319 (99.4) |  | 1180 (99.7) |  |
| Yes | 13 (0.6) |  | 3 (0.3) |  |
| Parity [N (%)] | | | | 0.584 |
| Nulliparous | 1986 (85.2) |  | 1128 (84.4) |  |
| Multiparous | 346 (14.8) |  | 208 (15.6) |  |
| Birth season [N (%)] | | | | **<0.001** |
| Spring | 445 (19.1) |  | 320 (27.9) |  |
| Summer | 661 (28.3) |  | 285 (24.9) |  |
| Autumn | 770 (33.0) |  | 368 (32.1) |  |
| Winter | 456 (19.6) |  | 172 (15.0) |  |
| Maternal Age (years) [Mean (SD)] | | | | **<0.001** |
|  | 28.5 (3.7) |  | 29.1 (3.7) |  |
| Gestational age (weeks) [Mean (SD)] | | | | **<0.001** |
|  | 39.0 (1.3) |  | 38.59 (2.4) |  |
| Birth weight (g) [Mean (SD)] | | | | 0.461 |
|  | 3372 (421) |  | 3356 (573) |  |
| Length (cm) [Mean (SD)] | |  |  | **<0.001** |
|  | 49.90 (1.2) |  | 49.45 (4.0) |  |
| Anogenital distance (mm) [Mean (SD)] | | | | 0.719 |
|  | 15.0 (5.5) |  | 15.1 (5.0) |  |

Abbreviations: SD: standard deviation.

**Table S2. Relationship between maternal exposure to PM_2.5_ (in quartiles) and offspring's** **anogenital distance**

| Male (N=1186) | β* | 95%CI | *P* value |  | Female (N=1146) | β* | 95%CI | *P* value |
| --- | --- | --- | --- | --- | --- | --- | --- | --- |
| Full pregnancy |  |  |  |  | Full pregnancy |  |  |  |
| Q1 (<46.0) | ref | ref | ref |  | Q1 (<46.3) | ref | ref | ref |
| Q2 (46.0~50.1) | -0.691 | -1.418, 0.036 | 0.063 |  | Q2 (46.3~50.4) | -1.486 | -2.158, -0.814 | **<0.001** |
| Q3 (50.1~53.0) | -0.975 | -1.872, -0.077 | **0.033** |  | Q3 (50.4~53.0) | -3.064 | -3.864, -2.263 | **<0.001** |
| Q4 (≥53.0) | -1.669 | -2.616, -0.723 | **<0.001** |  | Q4 (≥53.0) | -5.058 | -5.900, -4.217 | **<0.001** |
| P for trend | **<0.001** |  |  |  | P for trend | **<0.001** |  |  |
| First trimester |  |  |  |  | First trimester |  |  |  |
| Q1 (<41.4) | ref | ref | ref |  | Q1 (<43.0) | ref | ref | ref |
| Q2 (41.4~50.7) | -0.098 | -0.850, 0.653 | 0.797 |  | Q2 (43.0~52.1) | -1.610 | -2.357, -0.863 | **<0.001** |
| Q3 (50.7~63.7) | -0.812 | -1.720, 0.096 | **0.080** |  | Q3 (52.1~65.6) | -2.459 | -3.393, -1.525 | **<0.001** |
| Q4 (≥63.7) | 0.601 | -0.509, 1.710 | 0.289 |  | Q4 (≥65.6) | -2.419 | -3.559, -1.279 | **<0.001** |
| P for trend | 0.770 |  |  |  | P for trend | **<0.001** |  |  |
| Second trimester |  |  |  |  | Second trimester |  |  |  |
| Q1 (<38.8) | ref | ref | ref |  | Q1 (<39.0) | ref | ref | ref |
| Q2 (38.8~45.1) | -0.523 | -1.170, 0.124 | 0.1135 |  | Q2 (39.0~45.2) | -0.348 | -0.981, 0.285 | 0.224 |
| Q3 (45.1~57.3) | -1.385 | -2.199, -0.572 | **<0.001** |  | Q3 (45.2~55.6) | -1.418 | -2.189, -0.648 | **<0.001** |
| Q4 (≥57.3) | -1.907 | -2.988, -0.826 | **<0.001** |  | Q4 (≥55.6) | -2.079 | -3.081, -1.077 | **<0.001** |
| P for trend | **<0.001** |  |  |  | P for trend | **<0.001** |  |  |
| Third trimester |  |  |  |  | Third trimester |  |  |  |
| Q1 (<38.8) | ref | ref | ref |  | Q1 (<38.2) | ref | ref | ref |
| Q2 (38.8~45.2) | -0.622 | -1.262, 0.017 | 0.057 |  | Q2 (38.2~43.5) | -0.967 | -1.571, -0.363 | **0.002** |
| Q3 (45.2~56.6) | -1.726 | -2.511, -0.941 | **<0.001** |  | Q3 (43.5~56.5) | -1.873 | -2.615, -1.130 | **<0.001** |
| Q4 (≥56.6) | -1.069 | -2.118, -0.020 | **0.046** |  | Q4 (≥56.5) | -2.493 | -3.595, -1.392 | **<0.001** |
| P for trend | **<0.001** |  |  |  | P for trend | **<0.001** |  |  |

Abbreviations: PM_2.5_: particles with aerodynamic diameters of 2.5μm or less; 95% CI: 95% confidence interval.

*Multiple linear regression models were adjusted for maternal age, maternal education, pre-pregnancy BMI, maternal passive smoking during pregnancy, parity and birth season.

**Table S3. Sensitivity analysis for the relationship between maternal exposure to PM_2.5_ per 10μg/m^3^ and offspring's anogenital distance additionally adjusted for gestational age and birth weight.**

|  | β (95%CI) * | *P* value |
| --- | --- | --- |
| Male (N=1186) |  |  |
| Full pregnancy | **-2.03 (-2.68, -1.38)** | **<0.001** |
| First trimester | -0.16 (-0.47, 0.14) | 0.299 |
| Second trimester | **-0.74 (-1.07, -0.42)** | **<0.001** |
| Third trimester | **-0.62 (-0.96, -0.29)** | **<0.001** |
|  |  |  |
| Female (N=1146) |  |  |
| Full pregnancy | **-4.69 (-5.32, -4.07)** | **<0.001** |
| First trimester | **-0.86 (-1.18, -0.54)** | **<0.001** |
| Second trimester | **-1.12 (-1.46, -0.79)** | **<0.001** |
| Third trimester | **-1.39 (-1.71, -1.06)** | **<0.001** |

Abbreviations: PM_2.5_: particles with aerodynamic diameters of 2.5μm or less; 95% CI: 95% confidence interval.

*Multiple linear regression models were adjusted for maternal age, maternal education, pre-pregnancy BMI, maternal passive smoking during pregnancy, parity, birth season, gestational age and birth weight.

**Table S4. Sensitivity analysis for the relationship between maternal exposure to PM_2.5_ per 10μg/m^3^ and offspring's AGDs based on the data with multiple imputation.**

|  | Model 1* |  | Model 2* |  |
| --- | --- | --- | --- | --- |
|  | β (95%CI) | *P* value | β (95%CI) | *P* value |
| Male (N=1240) |  |  |  |  |
| Full pregnancy | **-1.70 (-2.15, -1.26)** | **<0.001** | **-2.01 (-2.66, -1.36)** | **<0.001** |
| First trimester | **0.27 (0.10, 0.44)** | **0.002** | -0.14 (-0.45, 0.17) | 0.370 |
| Second trimester | **-0.50 (-0.67, -0.33)** | **<0.001** | **-0.69 (-1.01, -0.37)** | **<0.001** |
| Third trimester | **-0.64 (-0.82, -0.46)** | **<0.001** | **-0.68 (-1.03, -0.34)** | **<0.001** |
|  |  |  |  |  |
| Female (N=1196) |  |  |  |  |
| Full pregnancy | **-2.01 (-2.45, -1.56)** | **<0.001** | **-4.52 (-5.14, -3.91)** | **<0.001** |
| First trimester | -0.16 (-0.32, 0.01) | 0.062 | **-0.81 (-1.12, -0.49)** | **0.002** |
| Second trimester | **-0.28 (-0.46, -0.10)** | **0.002** | **-1.00 (-1.32, -0.67)** | **<0.001** |
| Third trimester | **-0.42 (-0.59, -0.25)** | **<0.001** | **-1.53 (-1.87, -1.20)** | **<0.001** |

Abbreviations: PM_2.5_: particles with aerodynamic diameters of 2.5μm or less; 95% CI: 95% confidence interval.

*Model 1 was unadjusted; Model 2 was adjusted for confounders including maternal age, maternal education, pre-pregnancy BMI, maternal passive smoking during pregnancy, parity and birth season.

**Table S5. Sensitivity analysis for the relationship between maternal exposure to PM_2.5_ per 10μg/m^3^ and offspring's AGDs based on the data excluding** **low birth weight and high birth weight.**

|  | Model 1* |  | Model 2* |  |
| --- | --- | --- | --- | --- |
|  | β (95%CI) | P value | β (95%CI) | P value |
| Male (N=1054) |  |  |  |  |
| Full pregnancy | **-1.67 (-2.13, -1.20)** | **<0.001** | **-1.78 (-2.48, -1.08)** | **<0.001** |
| First trimester | **0.31 (0.13, 0.49)** | **<0.001** | -0.10 (-0.42, 0.22) | 0.535 |
| Second trimester | **-0.51 (-0.69, -0.32)** | **<0.001** | **-0.68 (-1.03, -0.33)** | **<0.001** |
| Third trimester | **-0.62 (-0.81, -0.43)** | **<0.001** | **-0.50 (-0.86, -0.15)** | **<0.001** |
|  |  |  |  |  |
| Female (N=1057) |  |  |  |  |
| Full pregnancy | **-2.05 (-2.52, -1.57)** | **<0.001** | **-4.71 (-5.37, -4.04)** | **<0.001** |
| First trimester | -0.12 (-0.30, 0.05) | 0.172 | **-0.75 (-1.08, -0.42)** | **<0.001** |
| Second trimester | **-0.26 (-0.45, -0.07)** | **0.010** | **-1.02 (-1.38, -0.66)** | **<0.001** |
| Third trimester | **-0.45 (-0.62, -0.27)** | **<0.001** | **-1.52 (-1.86, -1.19)** | **<0.001** |

Abbreviations: PM_2.5_: particles with aerodynamic diameters of 2.5μm or less; 95% CI: 95% confidence interval.

*Model 1 was unadjusted; Model 2 was adjusted for confounders including maternal age, maternal education, pre-pregnancy BMI, maternal passive smoking during pregnancy, parity and birth season.
