# Supplementary figures and images for: Prenatal exposure to fine particulate matter and newborn anogenital distance: a prospective cohort study
Source: Environ Health. 2023 Feb 9;22:16. doi: 10.1186/s12940-023-00969-w (PMC9909868; doi:10.1186/s12940-023-00969-w)

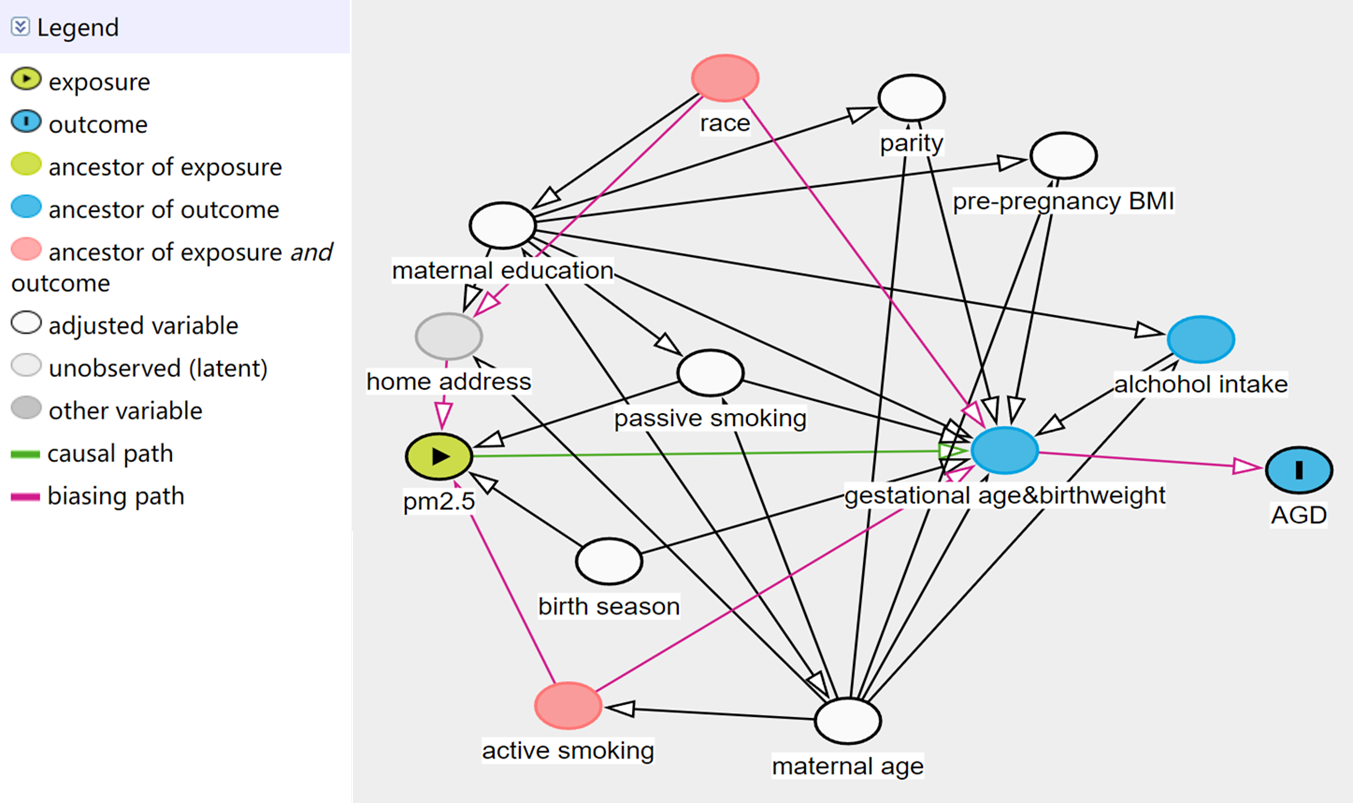


**Figure S1. Directed Acyclic Graph for covariates selection.**

Supplement: Supplementary file 2 — Additional file 2: Figure S1. Directed Acyclic Graph for covariates selection. [file 12940_2023_969_MOESM2_ESM.docx]
